# AP-1 Mediated Transcriptional Repression of Matrix Metalloproteinase-9 by Recruitment of Histone Deacetylase 1 in Response to Interferon β

**DOI:** 10.1371/journal.pone.0042152

**Published:** 2012-08-06

**Authors:** Megan L. Mittelstadt, Rekha C. Patel

**Affiliations:** Department of Biological Sciences, University of South Carolina, Columbia, South Carolina, United States of America; Northwestern University, United States of America

## Abstract

Matrix metalloproteinase-9 (MMP-9) is a 92 kDa zinc-dependant endopeptidase that degrades components of the extracellular matrix. Increased expression of MMP-9 is implicated in many pathological conditions including metastatic cancer, multiple sclerosis, and atherosclerosis. Although it has been widely noted that interferon-β (IFNβ) downregulates both the basal and phorbol 12-myristate 13-acetate (PMA)-induced MMP-9 expression at the transcriptional level, the molecular mechanism of this repression is poorly understood. In the present study we identify a novel mechanism for repression of MMP-9 transcription by IFNβ in HT1080 fibrosarcoma cells. Using reporter assays with promoter deletion constructs we show that IFNβ’s inhibitory effects require a region of the promoter between −154 and −72, which contains an AP-1 binding site. Chromatin immunoprecipitation (ChIP) studies indicate that IFNβ increases histone deacetylase (HDAC)-1 recruitment to the MMP-9 promoter and reduces histone H3 acetylation, in addition to reduced NF-κB recruitment. ChIP analysis shows that IFNβ induced HDAC1 recruitment to the MMP-9 promoter and IFNβ mediated transcriptional repression is lost when the AP-1 binding site is inactivated by a point mutation. Altogether, our results establish that the repression of MMP-9 transcription in response to IFNβ occurs by the recruitment of HDAC1 via the proximal AP-1 binding site.

## Introduction

Interferon-β (IFNβ) is a cytokine involved in antiviral, antiproliferative, and anti-angiogenic pathways [1]. IFNβactivates the janus kinase/signal transducer and activator of transcription (JAK/STAT) pathway which induces transcription of several genes via the formation of active transcription factor interferon-stimulated gene factor 3 (ISGF3). This heterotrimer translocates to the nucleus to bind to interferon-stimulated response elements (ISREs) in the promoters of IFNβ inducible genes, stimulating their transcription [2], [3]. In addition to transcriptional gene induction, IFNβ can also suppress the transcription of certain genes [4]. Although the signaling pathways and mechanisms leading to transcriptional induction by IFNs have been studied extensively, the mechanism of transcriptional downregulation by IFNs has not. IFNβ is used clinically for the treatment of multiple sclerosis and cancer [5]. One of the major mediators of IFNβ’s therapeutic actions is its ability to downregulate the expression of matrix metalloproteinase (MMP) family member MMP-9 [6]. IFNβ is known to repress MMP-9 at the transcriptional level, and acts to repress both basal and cytokine- or PMA-induced MMP-9 expression.

Regulation of MMP-9 is essential for various biological processes that include angiogenesis, inflammatory response, normal immune functions, differentiation of human embryonic stem cells, pregnancy and labor, and wound healing, etc [7], [8], [9], [10]. Enhanced expression of MMP-9 is involved in diverse pathological processes such as metastasis, tumor induced angiogenesis, and inflammatory conditions including rheumatoid arthritis, multiple sclerosis, lupus, and asthma [6], [8]. The elevated expression of MMP-9 in pathological contexts mainly occurs in response to either inflammatory or oncogenic signals. In cell culture systems, MMP-9 expression is also induced at the transcriptional level in response to various inflammatory stimuli such as lipopolysaccharides (LPS), interleukins, TNFα, and PMA. Several enhancer elements have been identified within MMP-9’s −670 bp promoter, including binding sites for NF-κB, Sp1, Ets, and AP-1. Two AP-1 binding sites (proximal and distal) have been shown to contribute to the transcriptional induction in response to various stimuli [11]. Of these sites, synergistic activation via the Sp1, NF-κB and proximal AP-1 site is required for full activation of the MMP-9 promoter by PMA in human fibrosarcoma cell line HT1080 [12].

In contrast to the amount of information available on the positive factors involved in transcriptional regulation of MMP-9, information on negative regulation of MMP-9 is limited. We have investigated the mechanism of IFNβ regulated transcriptional repression of MMP-9 promoter. Our results show that the proximal AP-1 binding site is essential for IFNβ’s ability to repress MMP-9 transcription. Furthermore, HDAC1 is recruited to the MMP-9 promoter in response to IFNβ treatment, dependent upon a functional proximal AP-1 site. We propose a model in which IFNβ leads to an increase in AP-1’s binding affinity for HDAC1 without a reduction in AP-1’s DNA binding capabilities. By specific recruitment of HDAC1 to the proximal AP-1 binding site within the MMP-9 promoter, AP-1 is able to act as a transcriptional repressor of MMP-9 under IFNβ treatment conditions. Our results establish that repression of MMP-9 transcription in response to IFNβ is primarily brought about by recruitment of HDAC1 to the proximal AP-1 site.

## Results

### Identification of the Promoter Element(s) Required for IFNβ Repression of MMP-9

**Figure 1:**
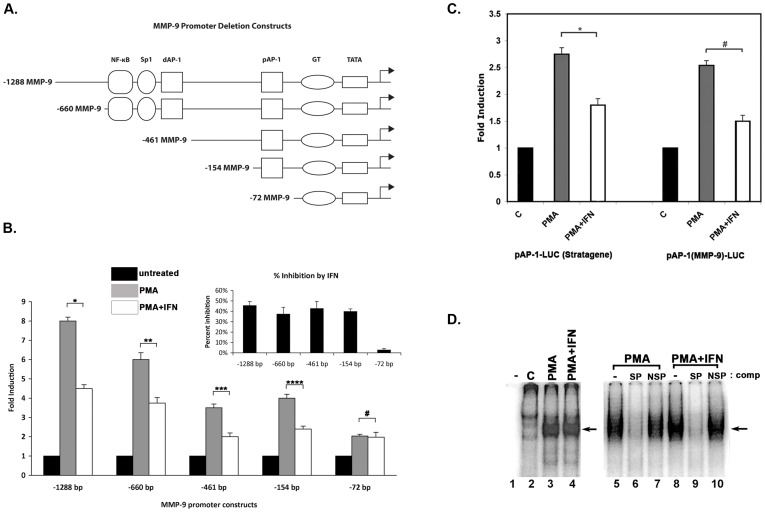
The -154 bp promoter construct retains the ability to respond to IFNβ. (A) Schematic diagram of the deletion constructs. Progressive deletions of MMP-9 promoter were created by PCR amplification with appropriate primers. Various transcription factor binding sites and the TATA box are as indicated. (B) Mapping the promoter region responsible for IFNβ mediated transcriptional repression. HT1080 cells were transiently transfected in 6 well plates in triplicates with MMP-9 promoter deletion constructs as indicated using Effectene transfection reagent (Qiagen). A plasmid encoding Renilla luciferase (pRL-null, Promega) was co-transfected for the normalization of transfection efficiencies. 24 hours after transfection, the cells were treated with 30 nM PMA and 100 U/mL IFNβ for 18 hours prior to preparation of cell extracts. Cell extracts were assayed for firefly and Renilla luciferase activities. Black bars indicate untreated values, considered as 1 for each construct. Grey bars indicate PMA treated values represented as fold inductions. White bars indicate PMA and IFNβ treated values also represented as fold inductions. The values represent averages of triplicate samples from four different experiments for each construct and the error bars represent standard deviation. The P-values were calculated using statistical analysis software. As indicated above bars, *, **, ***, and **** symbols indicate P values that indicate significant difference (0.0009, 0.0007, 0.0008, and 0.0009 resp.) and the symbol # indicates a P value of 0.1216 (no significant difference) with significant values being <0.01. Inset: The percent inhibition in response to IFNβ is plotted for each deletion construct. (C) AP-1 binding site is sufficient to confer downregulation by IFNβ on a synthetic reporter construct. HT1080 cells were co-transfected with either consensus AP-1 binding site concatamer (pAP-1-Luc, Stratagene) or MMP-9-specific AP-1 binding site concatamer (AP-1 (MMP9)-Luc) linked to a basal promoter-Luc, and a plasmid encoding Renilla luciferase (pRL-null, Promega). 24 hours after transfection, cells were treated with 30 ηM PMA and 100 U/mL IFNβ for 18 hours prior to preparation of cell extracts. Cell extracts were assayed for firefly and Renilla luciferase activities. Black bars indicate untreated cell values, considered as 1 for each construct. Grey bars indicate the PMA treated values represented as fold inductions. White bars indicate the PMA and IFNβ treated values represented as fold inductions. The values are averages from three separate experiments and the error bards represent standard deviation. The P-values were calculated using statistical analysis software. As indicated above the bars, *symbol indicates a P value of 0.0008 (significant difference) and the symbol # indicates a P value of 0.0001 (significant difference), with significant values being <0.01. (D) IFNβ does not inhibit the DNA-binding activity of AP-1. EMSA was carried out using the nuclear extracts prepared from HT1080 cells that were untreated or treated with PMA, PMA plus IFNβ for 30 minutes. 1 µg of nuclear extracts were incubated with the ^32^P-labeled AP-1 (region −93 to −56 of MMP-9 promoter) probe. Arrows indicate AP-1 complex bound to DNA. Unlabelled specific (SP: AP-1 consensus sequence) and non-specific (NSP: TATA box consensus sequence) competitor oligonucleotides in 100-fold molar excess were utilized to confirm the identity of the bound complex.

IFNβ is known to repress both basal and PMA induced MMP-9 at the transcriptional level [2], [13]. In order to determine the region of the MMP-9 promoter that is mediating IFNβ repression, a promoter deletion series inserted upstream of a firefly luciferase reporter (−1298 bp, −660 bp, −462 bp, −154 bp, and −72 bp) was utilized (Figure 1A). It was previously reported that the recruitment of IRF1 to a IFN-responsive-like element, which overlaps the NF-κB binding site at −615 bp, acts to partially repress MMP-9 expression in response to IFNγ due to IRF1’s competitive inhibition of NF-κB protein binding [13]. However, our results show that IFNβ mediated repression of MMP-9 (between 40% and 50% reduction in luciferase reporter activity, depending on size of MMP-9 promoter deletion) persists until the −154 bp deletion construct (Figure 1B), but is lost in the −72 bp construct. The poor induction of the −72 bp construct is not surprising as this construct does not retain the NF-κB, AP-1 and Sp1 binding sites in the promoter, which are known to be essential for PMA induction of the transcription. Of significance is the fact that the −461 bp and the −154 bp constructs retain strong repressive response to IFNβ (Figure 1B inset), thereby indicating that the loss of NF-κB site does not affect the ability of IFNβ to repress MMP-9 promoter. Based on these results, we concluded that the sequences present within the −154 bp construct are sufficient for mediating IFNβ repression on MMP-9 promoter. It should be noted that PMA induction is partially lost in the −461 bp and all other smaller constructs in the deletion series. This is expected, as PMA induction occurs due to synergistic activation through AP-1, NF-κB, and Sp1 transcription factors, and in the region between −661 and −461 bp of the MMP-9 promoter there exists an NF-κB, Sp1 and the distal AP-1 binding sites.

### The Proximal AP-1 Site within the MMP-9 Promoter Mediates Repression by IFNβ

Based on these results, we hypothesized that the proximal AP-1 binding site contained in the −154 bp deletion construct may be involved in mediating the downregulation of MMP-9 transcription in response to IFNβ. In order to determine whether the AP-1 binding site by itself is sufficient to confer transcriptional repression in response to IFNβ, we analyzed IFNβ’s effect on a synthetic construct regulated solely by a consensus AP-1 sequence (six repeats of sequence TGACTAA, Stratagene). HT1080 cells were transfected with pAP-1-Luc (Stratagene), and promoter activity in response to PMA or PMA plus IFNβ was determined. As shown in figure 1C, this construct showed a 2.75-fold induction of luciferase activity in response to PMA, followed by 37% repression in response to IFNβ plus PMA treatment (Figure 1C). To further examine IFNβ’s inhibition on AP-1 activity, we constructed a synthetic luciferase reporter construct regulated solely by six repeats of the proximal AP-1 site as it exists within the MMP-9 promoter (TGAGTCA). HT1080 cells were transfected with pAP-1(MMP9)-Luc, and promoter activity in response to PMA or PMA plus IFNβ was determined. In response to PMA, pAP-1(MMP9)-Luc showed a 2.5-fold induction while in response to PMA plus IFNβ, pAP-1(MMP9)-Luc showed 40% repression (Figure 1C). These results indicate that the AP-1 binding site is sufficient to allow induction in response to PMA. More importantly, our results establish that a consensus AP-1 site can also mediate transcriptional repression by IFNβ, at a level consistent with the amount of IFNβ-mediated repression seen in the full length MMP-9 promoter.

### IFNβ does not Affect the Binding of AP-1 to the MMP-9 Promoter

To examine whether IFNβ brings about the repression of MMP-9 transcription by inhibiting the binding of AP-1 to the proximal promoter region, we utilized EMSA analysis. Nuclear extracts were isolated from HT1080 cells and used to detect AP-1 binding to an oligonucleotide containing the proximal AP-1 sequence within the MMP-9 promoter and a few nucleotides flanking this site. As shown in figure 1D, nuclear extracts from PMA treated cells showed an induction of a binding activity as indicated by an arrow (lane 3), compared to the extracts from untreated cells (lane 2). There was no change detected in this mobility-shifted complex when nuclear extracts from cells treated with PMA and IFNβ were utilized (lane 4). Thus, the *in vitro* DNA binding capability of AP-1 was unchanged in response to IFNβ treatment as compared to the extracts isolated from PMA treated cells. In order to confirm that the observed mobility-shifted protein complex is indeed the AP-1 complex, unlabelled oligonucleotides in 100-fold molar excess were used as specific and non-specific competitors during the binding step. An AP-1 consensus oligonucleotide used as a competitor abolished the presence of AP-1-DNA complex in both PMA and PMA plus IFNβ samples (Figure 1D, lanes 6 and 9), thereby confirming that the complex as AP-1. A TFIID binding consensus oligonucleotide used as a nonspecific competitor (lanes 7 and 10) did not disrupt the binding of the AP-1 to the probe, further confirming the specificity of the AP-1 complex. These data indicate that IFNβ does not repress MMP-9 transcription by rendering AP-1 inactive for binding to the proximal AP-1 site. This is in accordance with data previously reported by Zhao et al: recruitment of c-fos and JunD subunits of AP-1 to the MMP-9 promoter is increased under PMA treatment conditions, but does not decrease under IFNβ treatment conditions [2].

### Binding of AP-1 to the Proximal Promoter Region of MMP-9 is Required for IFNβ Mediated Repression

**Figure 2:**
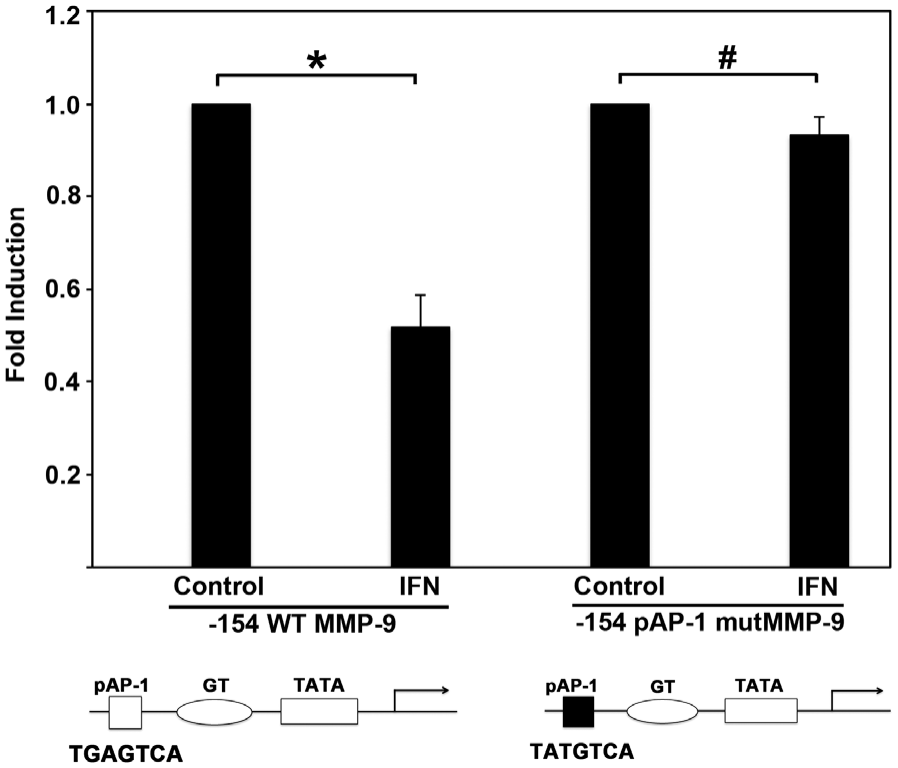
Loss of IFNβ mediated repression of MMP-9 promoter when the binding of AP-1 to the proximal site is abrogated. HT1080 cells were transfected with −154 MMP-9 promoter deletion construct containing a point mutation in the proximal AP-1 site, which renders the binding site non-functional. Cells were prepared as described previously and were treated with 100 U/mL IFNβ for 18 hours prior to preparation of cell extracts. Cell extracts were assayed for firefly luciferase activities, and normalized by protein concentration to account for any cell concentration differences between samples. Black bars: untreated values considered as 1 for each construct, white bars: IFNβ treated. The experiment was repeated three times and the error bars represent standard deviation calculated from three separate experiments. The P-values were calculated using statistical analysis software. As indicated above the bars, * symbol indicates a P value of 1.8×10^−4^ (significant difference) and the symbol # indicates a P value of 0.092 (no significant difference), with significant values being <0.01.

Since AP-1 is sufficient to confer IFNβ mediated repression on a heterologous promoter (Figure 1C) and IFNβ did not prevent AP-1’s DNA binding ability in response to IFNβ (Figure 1D, and [14], we next examined whether AP-1 binding could in fact be required for IFNβ mediated transcriptional repression of MMP-9 promoter. An MMP-9 expression construct was utilized which contained a mutated proximal AP-1 site, and had been previously described to disrupt binding of AP-1 subunits, rendering the AP-1 binding site non-functional [15]. This construct was transfected into HT1080 cells alongside the wt −154 bp MMP-9 construct and luciferase activities were compared after treatment with IFNβ. Promoter activity of the wt −154 bp promoter was downregulated 45% under IFNβ treatment conditions, similar to the amount of repression seen in the −660 bp promoter (Fig. 1 B) under IFNβ treatment conditions. Importantly, this repression is nearly absent in the −154 bp promoter containing the AP-1 site point mutation (Figure 2). Treatment with PMA does not result in induction of the −154 AP-1 mutant promoter piece, as this promoter segment does not contain any of the *cis*-acting binding sites known to mediate PMA induction of MMP-9 (NF-κB, Sp1, or AP-1) (data not shown). Because of this striking alteration in the capacity for downregulation under IFNβ treatment conditions, we concluded that binding of AP-1 to the proximal AP-1 binding site of the MMP-9 promoter is essential for IFNβ repression. Additionally, because the binding of AP-1 is required for IFNβ repression, it is possible that AP-1 may be recruiting a repressor to the MMP-9 promoter under IFNβ treatment conditions to bring about downregulation of MMP-9 transcription.

### Proximal Promoter Containing the AP-1 Binding Site Mediates HDAC Involvement

**Figure 3:**
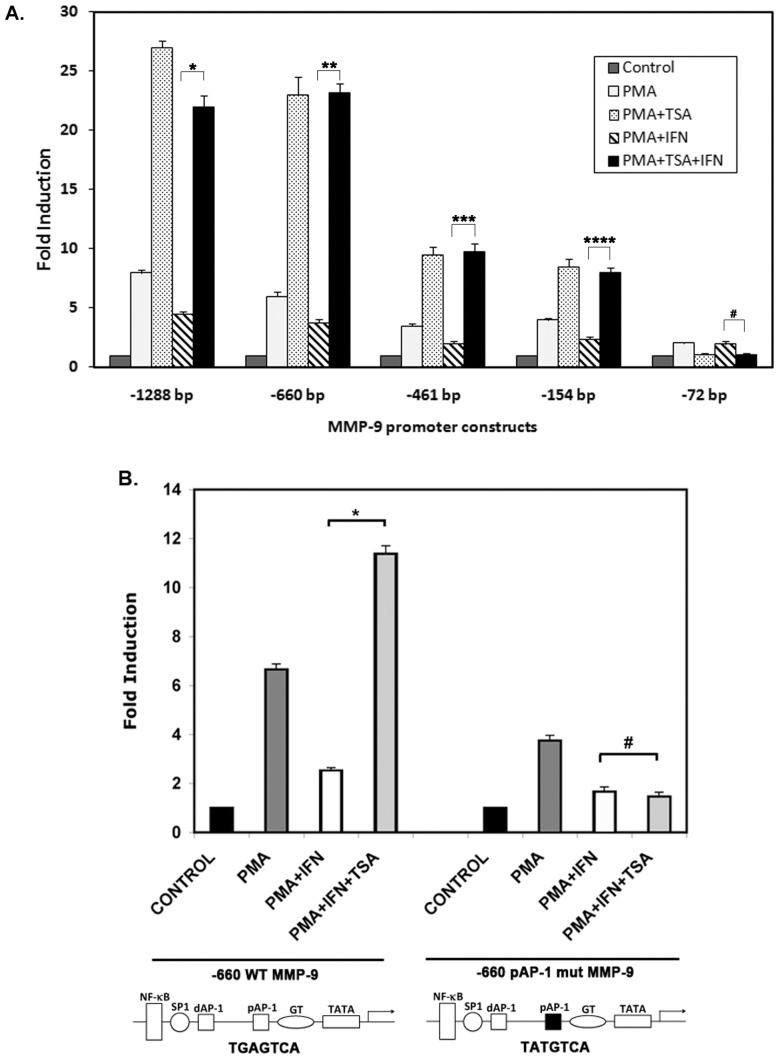
Involvement of HDAC in IFNβ mediated repression of MMP-9 promoter. (A) Response of various promoter deletion constructs to a HDAC inhibitor. HT1080 cells were transiently co-transfected with MMP-9 promoter constructs. 24 hours after transfection, cells were pooled and redistributed amongst the wells, so that transfection efficiency was the same for all samples. 48 hours after transfection, the cells were treated with 220 nM TSA, 30 nM PMA and 100 U/mL IFNβ as indicated for 18 hours prior to preparation of cell extracts. Cell extracts were assayed for firefly luciferase activities, and normalized by protein concentration to account for any cell concentration differences between samples. Grey bars: untreated, white bars: PMA treated, grey stippled bars: PMA + TSA, hatched bars: IFNβ, and black bars: PMA + IFNβ + TSA. The P-values were calculated using statistical analysis software. As indicated above bars, *, **, ***, and **** symbols indicate P values that indicate significant difference (0.0011, 0.0009, 0.0007, and 0.0008 resp.) and the symbol # indicates a P value of 0.0874 (no significant difference) with significant values being <0.01. (B) AP-1 binding to the proximal site is required for HDAC1 recruitment to MMP-9 promoter in response to IFNβ. HT1080 cells were transfected in 6-well dishes in triplicates with −660 MMP-9 promoter deletion construct containing a point mutation in the proximal AP-1 site, which renders the binding site non-functional, or with wild type −660 MMP-9 construct. Cells were prepared as described previously, including treatments with 220 nM TSA, 30 nM PMA and 100 U/mL IFNβ for 18 hours prior to preparation of cell extracts. Cell extracts were assayed for firefly luciferase activities, and normalized by protein concentration to account for any cell concentration differences between samples. Black bars: untreated values considered as 1 for each construct, dark grey bars: PMA treated, white bars: PMA + IFNβ, and light grey bars: PMA + IFNβ + TSA. The experiment was repeated three times and the error bars represent standard deviation calculated from three separate experiments. The P-values were calculated using statistical analysis software. As indicated above the bars, * symbol indicates a P value of 0.0004 (significant difference) and the symbol # indicates a P value of 0.47 (no significant difference), with significant values being <0.01.

It has been demonstrated that metastasis associated gene MTA1, which associates with the NuRD complex, reduces both basal and PMA induced MMP-9 protein and mRNA levels in HT1080 cells. MTA1’s ability to repress MMP-9 is partially dependent upon HDAC2 recruitment by MTA1 and reduced histone H3 and H4 acetylation at the MMP-9 promoter [11]. In order to investigate if HDAC recruitment could be a possible mechanism of repression of MMP-9 transcription by IFNβ, we utilized a broad spectrum HDAC inhibitor, trichostatin A (TSA). Strikingly, our results show that TSA treatment rescued the IFNβ mediated downregulation of PMA induced MMP-9 transcription in −1288 bp through −154 bp promoter constructs (Figure 3A). Both IFNβ mediated downregulation as well as the TSA rescue of MMP-9 transcription is lost in the −72 bp deletion construct (Figure 3A). Since the −154 bp construct retains the capacity to respond to TSA mediated rescue, the region between −154 and −72 bp mediates HDAC involvement in transcriptional regulation of MMP-9. Thus, the proximal AP-1 site located within this region may mediate the recruitment of HDAC.

We also transfected cells with the wild type −660 bp construct and the −660 proximal AP-1 site mutant construct into HT1080 cells. Luciferase activity was compared after PMA and IFNβ treatment. PMA induction is reduced in the AP-1 mutant as compared to the wild type due to loss of AP-1 binding but significant PMA induction is still observed (Figure 3B). IFNβ is still able to exert a partial repressive effect on the −600 mutant construct. This data is in agreement with another group’s finding that under IFNγ treatment conditions, IRF-1 binds to the MMP-9 promoter at a location that overlaps the MMP-9 promoter’s NF-κB binding site, competing with the binding of transcription factor NF-κB [13]. However, because our data also show that IFNβ is still fully able to repress MMP-9 after deletion of the NF-κB binding site (Figure 1B), we hypothesize that a reduction in the binding efficiency of NF-κB is likely secondary to the mechanism mediated by IFNβ through the proximal AP-1 binding site. Most importantly, the wild type −660 bp MMP-9 promoter responds to TSA treatment with the rescue of IFNβ mediated repression (Figures 3A and 3B), but in the proximal AP-1 site point mutated promoter, the rescue of IFNβ repression in response to TSA is lost. Therefore, a functional proximal AP-1 binding site is required for rescue of IFNβ repression of MMP-9 by HDAC inhibition. While IRF-1 binding competition with NF-κB may be induced by IFNγ treatment, these results point strongly to the AP-1 binding site as another major mediator of IFNβ repression of MMP-9 transcription.

### Recruitment of HDAC1 to the MMP-9 Promoter during Transcriptional Repression by IFNβ

**Figure 4:**
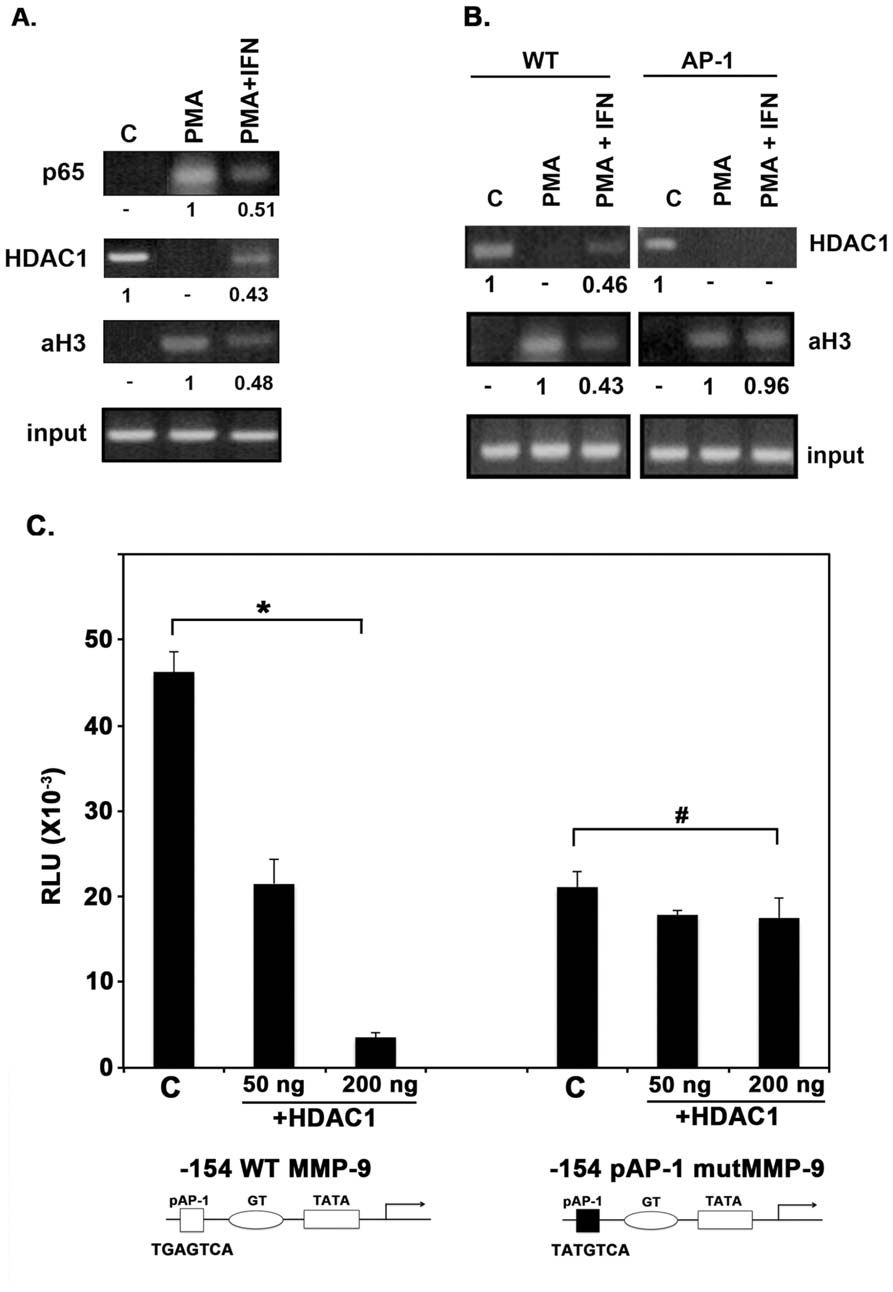
Chromatin Immunoprecipitation (ChIP) analysis of the MMP-9 promoter. (A) HDAC1 recruitment on MMP-9 promoter in response to IFNβ. HT1080 cells were treated with IFNβ for 12 hrs, PMA for 4 hrs, or both. ChIP assays, using antibodies specific to p65, histone deacetylase (HDAC)-1, or acetylated histone-3 (aH3) were performed, followed by PCR performed within the linear range of amplification (determined to be 38 cycles). Input chromatin (1%) was removed from samples prior to immunoprecipitation and subjected to PCR to control for any variation in starting material. Band intensities were normalized to input and the relative band intensities were calculated. The relative band intensities are shown under each band with respect to the strongest band being represented as 1. Data shown is representative of four independent experiments. (B) HDAC1 recruitment on MMP-9 promoter in response to IFNβ is prevented in vivo by rendering AP-1 site non-functional. HT1080 cells were stably transfected with −660 bp MMP-9 promoter luciferase reporter constructs, either with or without proximal AP-1 site mutated. Resulting stable lines were treated with IFNβ for 12 hrs, PMA for 4 hrs, or both. Chromatin immunoprecipitation (ChIP) assays, using antibodies specific to histone deacetylase (HDAC)-1, or acetylated histone-3 (aH3) were performed, followed by PCR. PCR primers were designed to include the region of interest of the MMP-9 promoter and a section of the luciferase reporter, to select against amplification of endogenous wild type MMP-9 promoter region. Band intensities were normalized to input and the relative band intensities were calculated. The relative band intensities are shown under each band with respect to the strongest band being represented as 1. Data shown is representative of three independent experiments. (C) AP-1 binding to the proximal site is required for HDAC1 induced repression of MMP-9 HT1080 cells were co-transfected in 6-well dishes in triplicates with either −154 MMP-9 promoter deletion construct containing a point mutation in the proximal AP-1 site, which renders the binding site non-functional, or with wild type −154 MMP-9 construct and varying amounts of a HDAC1 expression construct. Cell extracts were assayed for firefly luciferase activities, and normalized by protein concentration to account for any cell concentration differences between samples. P-values were calculated using statistical analysis software. As indicated above the bars, * indicates P-values of significance (50 ng  =  0.000698 and 100 ng  =  0.000885, significant difference) and # indicates P-values of no significance (50 ng  =  0.0763 and 100 ng  =  0.882, no significant difference) with significant values being <0.01.

Although it is known that transiently transfected DNA acquires immediate nucleosomal assembly on the plasmid, it is likely that the placement of histones along the endogenous gene is structurally dissimilar to that of the transiently introduced gene [16]. To circumvent this, the endogenous protein occupation status of the MMP-9 promoter was studied using chromatin immunoprecipitation (ChIP) assay. ChIP assays were performed using antibodies against p65 subunit of NF-κB, HDAC1, and acetylated histone H3. PCR analysis was used to probe immunoprecipitated samples for the presence of the MMP-9 promoter region surrounding the proximal AP-1 binding site (primers were designed to amplify the region of interest between −154 bp and +20 bp). PCR analysis of the control (input) indicated that the soluble chromatin samples obtained from each treatment condition contained equal amounts of chromatin fragments of the MMP-9 promoter region of interest (Figure 4A, input bands). In untreated cells, p65 association with the MMP-9 promoter was not detectable. As expected, in PMA treated cells, p65 shows a strong association with the MMP-9 promoter, corresponding to the requirement of NF-κB activation and binding at the −600 region of the promoter for transcriptional induction of MMP-9 in response to PMA. However, in cells treated with both PMA and IFNβ, p65 association with the MMP-9 promoter is significantly decreased. Thus, similar to IFNγ, it seems likely that IFNβ also induces binding of IRF1 to a site which competes with NF-κB’s binding at the MMP-9 promoter [13]. HDAC1 contributes to general histone deacetylation and condensed chromatin, so it is not surprising that there is a basal level of association of HDAC1 with the MMP-9 promoter under uninduced conditions (Figure 4A). In PMA treated cells, HDAC1 association with the MMP-9 promoter is undetectable; but with the addition of IFNβ, HDAC1 is recruited back to the proximal region of the MMP-9 promoter. Concurrently, the acetylation status of the MMP-9 proximal promoter region is reflective of the amount of HDAC1 present: as HDAC1 association is diminished, acetylation of histone H3 is increased and when HDAC1 returns to the MMP-9 promoter under IFNβ treatment, acetylation of histone H3 is decreased. Thus, histone H3 acetylation increases with PMA treatment and then decreases with IFN treatment (Figure 4A). It is likely that in addition to HDAC1, other histone deacetylases are also recruited to the MMP-9 proximal promoter region in a very similar manner. At this point, we have not examined involvement of other HDAC family members in regulation of MMP-9 promoter in response to IFNβ.

### Proximal AP-1 Binding Site is Required for Recruitment of HDAC1 Under IFNβ Treatment

To address the role of the proximal AP-1 binding site in recruitment of HDAC1 to MMP-9 promoter in response to IFNβ treatment *in vivo*, we created stably transfected HT1080 cells containing either the wt MMP-9 −660 bp promoter or MMP-9 −660 bp proximal AP-1 mutant promoter. Seven independent stably transfected clones for each construct were tested for luciferase activity to ensure that the random insertion point of the MMP-9 promoter did not affect PMA induction (in both the wild type and mutant clones) or IFNβ repression and TSA rescue of IFNβ repression (in the wild type clones). One wild type and one proximal AP-1 mutant clone were selected for ChIP assay analysis. ChIP analysis was performed under the same conditions as previously described, except that PCR primers were used which amplified the transgene-encoded transcript (by the use of a luciferase-specific downstream primer) and not the endogenous MMP-9 transcript. As shown in figure 4B, the wild type stably transfected cells responded to treatment as shown previously with endogenous MMP-9 in normal HT1080 cells (Figures 4A and 4B). However, the cells containing the AP-1 mutant promoter were not able to recruit HDAC1 to the proximal promoter region after IFNβ treatment. Concordantly, acetylation of histone H3 is mostly unchanged on AP-1 mutant promoter under IFNβ treatment. These results establish that HDAC1 recruitment to the MMP-9 promoter in response to IFNβ requires a functional AP-1 binding site.

To further confirm that recruitment of HDAC1 via the proximal AP-1 site is crucial for IFNβ mediated repression of MMP-9, we co-transfected either the wild type or mutant AP-1 −154 bp MMP-9 promoter construct along with 50 ng or 200 ng of HDAC1 expression construct (Figure 4C). We found there to be a dose-dependent downregulation of MMP-9 promoter activity in the presence of ectopic HDAC1 expression (also seen in Fig. S1A). However, neither 50 ng nor 200 ng HDAC1 was able to exert a repressive effect on the MMP-9 promoter containing a mutated proximal AP-1 site (Figure 4C). This reinforces the specific role of HDAC1 recruitment to the proximal AP-1 site as a co-repressor to downregulate transcription of MMP-9.

### HDAC1 is a Negative Regulator of MMP-9 Transcription

The effect of HDAC1 overexpression on MMP-9 induction was tested to further ascertain the repressive actions of HDAC1. We overexpressed, in increasing amounts, HDAC1 expression construct in HT1080 cells by transient co-transfection with −1.2 kb MMP-9 promoter reporter construct, and assayed for the luciferase activity. We tested the effect of HDAC1 overexpression on both the basal as well as PMA induced levels of MMMP-9 promoter activity. Results show that overexpression of HDAC1 downregulated both basal and PMA induced MMP-9 promoter activity in a dose-dependent manner (Figure S1A). This data further confirms that HDAC1 can regulate MMP-9 transcription negatively.

We reasoned that if HDAC1 is responsible for repression of MMP-9 promoter activity in response to IFNβ, an overexpression of a histone acetyl transferase such as p300 may be able to rescue the IFNβ mediated repression. Thus, we tested the effect of co-transfection with a p300 expression construct on the ability of IFNβ to repress the promoter activity in the −660 bp construct. Indeed, when the −660 bp MMP-9 promoter construct is co-transfected with 200 ng of p300, MMP-9 promoter activity is no longer downregulated under IFNβ treatment conditions (Figure S1B). The p300 expression construct could rescue IFNβ’s repressive actions in a dose dependent manner with the optimal rescue occurring at 200 ng (data not shown). CBP/p300 may act to oppose HDAC1 by increasing general histone acetylation at the MMP-9 promoter, or by acting as a cofactor for the assembly of NF-κB or AP-1 complexes at the MMP-9 promoter, or both [17].

### A Schematic Model for IFNβ Mediated Transcriptional Repression of MMP-9 Expression

**Figure 5:**
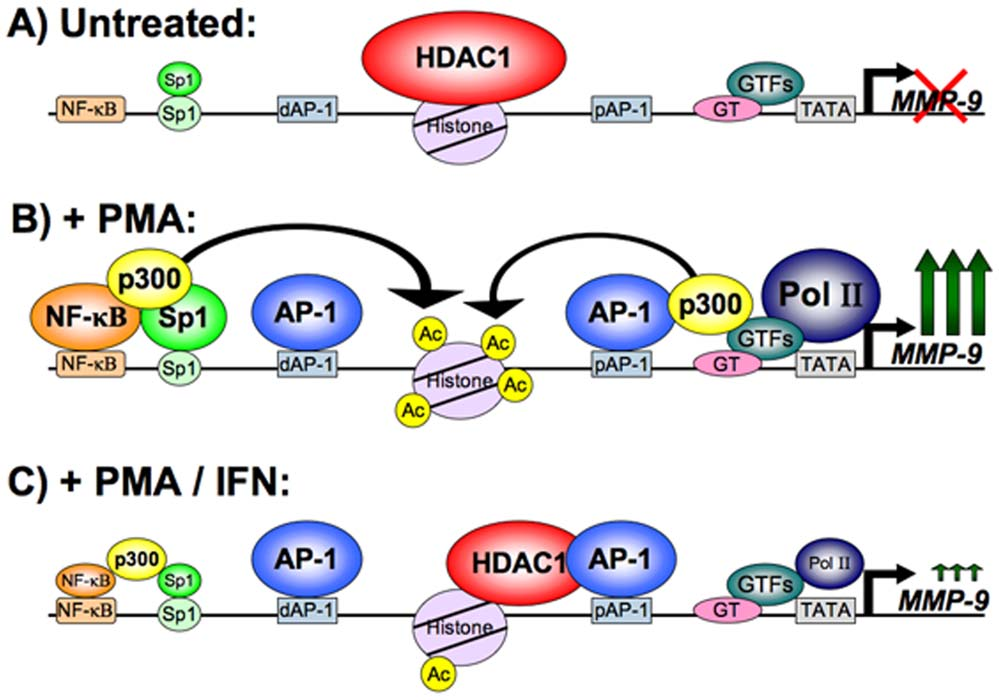
AP-1-mediated recruitment of HDAC1 to MMP-9 promoter in response to IFNβ - a schematic model. Based on our results and some of the previously published work [18], this model indicates what is occurring at the MMP-9 promoter under IFNβ treatment conditions. (**A**) Under uninduced conditions, there is a basal level of HDAC1 and general transcription factor occupation of the MMP-9 promoter. (**B**) Upon PMA treatment, recruitment of specific transcription factors NF-κB, AP-1, and Sp1 occurs and MMP-9 transcription is induced. Coactivators are also recruited, such as p300, which results in acetylation of the lysine tails of histones. (**C**) IFNβ treatment causes HDAC1 to be recruited to the proximal AP-1 site and a corresponding decrease in local acetylation, leading to decreased transcriptional activation of MMP-9. While AP-1 occupation of the MMP-9 promoter is unchanged under IFNβ treatment, NF-κB and other specific activators leave the promoter.

Our results describe a mechanism whereby transcription factor AP-1 acts to downregulate transcription of MMP-9 gene in contrast to its usual role as a transcription factor. Based on our results and previously published work [18] we propose a model for the mechanism of IFNβ mediated transcriptional downregulation of MMP-9 promoter. Under uninduced conditions, general transcription factors occupy the MMP-9 promoter along with HDAC1, contributing to a tight chromatin structure which excludes RNA polymerase binding (Figure 5, panel A). Under induced conditions NF-κB, AP-1, other cis-acting transcription factors, and co-activators such as p300 are recruited to the MMP-9 promoter. There is an increased local histone acetylation, which confers a loose chromatin structure, binding of RNA polymerase and high transcriptional activation of MMP-9 (Figure 5, panel B). Under IFNβ treatment conditions, NF-κB occupation of the MMP-9 promoter is decreased and HDAC1 is recruited via the proximal AP-1 site to the MMP-9 promoter. The DNA binding of AP-1 is unchanged under IFNβ treatment (Figure 5, panel C). We propose that the recruitment of HDAC1 on MMP-9 promoter occurs via interaction with the AP-1 transcription factor, and that certain posttranslational modification(s) of AP-1 that occurs in response to IFNβ mediates this interaction. Consequently, there is a decreased local histone acetylation after IFNβ exposure, which confers a more tightly packed chromatin structure and may result in reduced transcription of MMP-9.

## Discussion

MMP-9 is a 92 kDa type IV collagenase that belongs to the gelatinase group of secreted MMPs. Once activated, MMP-9 can interact with one of its substrates, which include but are not limited to: gelatin, elastin, laminin, fibronectin, myelin, collagen (types 4, 5, 7, 10, 11, and 17), serine protease inhibitors, galactoside binding proteins, and pro-tumor necrosis factor (TNF)-α [12]. Normally, MMP-9 is expressed at a low level in many cell types and while tight control of MMP-9 activity is essential for normal development, abnormal expression of MMP-9 contributes to disease processes such as tumor growth and metastasis.

Repression of MMP-9 is an integral part of IFNβ’s efficacy as a therapeutic for multiple sclerosis [19]. Until now, the mechanism of repression of MMP-9 by IFNβ had not been completely elucidated. Zhao et al demonstrated that all three proteins that constitute the IFNβ-activated interferon-stimulated gene factor 3 (ISGF3) complex (STAT-1, STAT-2 and interferon regulatory factor 9) are required for IFNβ mediated transcriptional inhibition of MMP-9 expression [2]. Furthermore, their data indicated that IFNβ reduces the recruitment of transcriptional activators and coactivators, such as NF-κB, Sp1, CREB-binding protein and p300, to the MMP-9 promoter, and decreases the histone acetylation at the MMP-9 promoter in the absence of an association of the ISGF3 complex with the MMP-9 promoter. Ma et al reported that IFNγ suppresses the MMP-9 transcription in a STAT-1 dependent manner by sequestration of coactivator CBP [18]. In another study, the class II major histocompatibility complex transactivator (CIITA) was implicated in sequestration of CBP in response to IFNγ, thereby making it unavailable at the MMP-9 promoter consequently reducing transcription [20]. Sanceau et al reported that the transcriptional repression of MMP-9 by IFNγ occurred primarily by inhibition of NF-κB binding to the MMP-9 promoter due to binding of interferon regulatory factor-1 (IRF1) to an overlapping site in response to IFN treatment [13]. In view of these multiple reported mechanisms, we investigated repression of MMP-9 transcription in human fibrosarcoma cell line HT1080. Our data demonstrate that the proximal AP-1 site is required for the repression of MMP-9 transcription, and that HDAC1 is recruited to the proximal AP-1 site in response to IFNβ. This is a novel mechanism whereby the transcription factor AP-1 can act as a negative factor presumably by virtue of its interaction with HDAC1 only under IFNβ treatment conditions.

There is some evidence that members of the IFN family affect AP-1 expression and/or activation. IFNγ suppresses transcription of interleukin (IL)-10 by reducing the expression of c-fos and by also reducing the transcription and nuclear accumulation of c-jun. However, unlike our studies with MMP-9 promoter and IFNβ, IFNγ was shown to suppress IL-10 expression by reducing AP-1’s DNA binding activity [21]. AP-1 family member c-jun has also been shown to interact with HDAC3 to repress transcription of target promoters. The induction of AP-1 mediated transcription was shown to occur as a result of a dissociation of an inhibitory complex that included HDAC3 in response to c–jun phosphorylation by JNK at serines 63/73 and at threonines 91/93. In this study, c-jun mediated transcriptional activation was thought to result from ‘activation by de-repression’ and this was primarily brought about by phosphorylation-dependent release of HDAC3 interaction with c-jun [22]. Additionally, Drosophila homologs of the AP-1 family have previously been shown to interact with HDACs. Regulation of the Drosophila immune response occurs through crosstalk between the JNK and Iκκ pathways. In Drosophila, binding of dAP-1 to the promoters of genes activated by the NF-κB homolog Relish led to recruitment of dHDAC1, followed by local modification of histone acetylation. This mechanism allows AP-1 to terminate the activation of a group of NF-κB target genes [23], [24]. Although there is evidence of interaction between AP-1 and HDAC family members, our work is the first example of IFNβ inducing the recruitment of HDAC1 by AP-1 to directly repress expression at the transcriptional level. Collectively, the results presented here not only confirm but also significantly extend previous results concerning the mechanism of transcriptional repression of MMP-9 in response to IFNβ.

We show that the repression of MMP-9 transcription in response to IFNβ occurs through recruitment of HDAC1 to the proximal AP-1 site. Since we specifically investigated only one member of the class I HDAC family, it is entirely possible that additional members of class I HDACs can interact with the proteins that bind to the AP-1 binding site. There is especially potential for HDAC2, which often binds to complexes alongside HDAC1; and for HDAC3, which is known to interact both with the MMP-9 promoter and also with AP-1 subunit c-jun [22], [25]. Whether the interaction between AP-1 subunits and HDAC1 is a direct one or whether additional binding partners mediate the interaction has yet to be established. The identification of AP-1 family members that mediate the interaction and the post-translational modifications that induce an increased binding affinity to HDAC1 in response to IFNβ remains to be investigated in future. Binding of HDAC1 to MMP-9 promoter affects the acetylation status of local histones (figure 4A), but could also affect the acetylation status and activation of other proteins. Several transcription factors such as PTEN, APEX1, and NF-κB are known to have altered transcriptional activation capabilities in regards to their acetylation status, and have all been shown to respond to class I HDACs [26], [27], [28]. Thus, the recruitment of HDAC1 to MMP-9 promoter may also contribute via its effect on NF-κB activity.

Because MMP-9 is a fundamental player in the cell migration and metastasis of numerous pathological contexts, an evaluation of the currently popular HDAC inhibitor class of therapeutics should be considered in view of our results. Though certain broad-spectrum HDAC inhibitors are currently being utilized for the treatment of cancers (Vorinostat/SAHA), the mechanism of action for HDAC inhibitors as cancer therapeutics is not completely clear. That is, while HDAC inhibitors undoubtedly work to restore expression of tumor-suppressors and other genes that are detrimental to the cancer, these inhibitors are quite likely to activate a variety of other genes that could promote the progression of the cancer. Perhaps this effect is negligible in some cancer contexts, but because we show that HDAC inhibitors contribute to the overexpression of MMP-9, HDAC inhibitors may also have the capability of enriching a subpopulation of highly metastatic cancer cells especially if a few of the cancer cells escape being eliminated in the first rounds of chemotherapy.

In conclusion, we have demonstrated that the proximal AP-1 binding site mediates the repression of MMP-9 by IFNβ. Upon IFNβ treatment, HDAC1 is recruited to the proximal AP-1 site of the MMP-9 promoter, contributing to a reduction in local histone acetylation and consequently reducing transcription. Our study suggests that some post-translational modification of AP-1 that occurs in response to IFNβ may result in its increased binding affinity to HDAC1, thereby switching the AP-1 transcription factor complex from being an essential transcription factor to a negative factor that is required for the repression of transcription in response to IFNβ.

## Materials and Methods

### Reagents and Plasmids

Phorbol 12-myristate 13-acetate (Sigma) was used at a final concentration of 30 nM. Human IFNβ (BioSource Int.) was used at a final concentration of 100 U/mL. Trichostatin A (T8552, Sigma) was used at a final concentration of 220 nM. 1.2 kb human MMP-9 promoter and progressive deletion mutants from the 5′ end of the promoter were created and cloned into pGL2Basic plasmid (Promega) upstream of a firefly luciferase reporter gene. PCR amplification deletion endpoints were generated to the following locations relative to the transcription start site: −1285 (5′-GCGGTACCGGGAGGGAGGCTTGGCATAA-3′), −660 (5′-GCGGTACCTACTGTCCCCTTTACTGCCCTGAA-3′), −461 (5′-GCGGTACCTCAAAGAAGGCTGTCAGC-3′), −154 (5′-GCGGTACCGCCCTTTCTCATGCTGGTGCTGCC-3′), and −72 (5′-GCGGTACCGCACTTGCCTGTCAAGGAGG-3′) and MMP-9 downstream primer (5′-GCCTCGAGTGGTGAGGGCAGAGGTGTCT-3′). The primer design for PCR was such that each upstream primer included a KpnI site and the downstream primer included a XhoI site. A synthetic construct containing the consensus sequence for AP-1 binding, pAP-1-Luc (seven repeats of TGACTAA), was purchased from Stratagene. An insert was also designed containing six repeats of MMP-9 specific AP-1 binding site (TGAGTCA), which we then inserted into the same luciferase construct that contained the consensus sequence (p-LUC-MCS, Stratagene), utilizing the restriction site, PstI, which flanked the consensus insert. MMP-9 promoter mutant was a kind gift from Dr. Ernst Lengyel, University of Chicago. This construct included the AP-1 site mutated from TGAGTCA to T**AT**GTCA. p300 expression construct was a kind gift from Chandrashekhar Patel, University of South Carolina School of Medicine. Flag-tagged HDAC1 expression construct was a kind gift from Dr. Edward Seto, H. Lee Moffitt Cancer Center.

### Cell Culture

HT1080 fibrosarcoma cells [29] were a kindly provided by George Stark, Cleveland Clinic Lerner Research Center and were maintained in Dulbecco’s modified Eagle’s medium (DMEM) supplemented with 100 U/mL penicillin, 100 µg/mL streptomycin and 10% fetal bovine serum. MMP-9 luciferase WT and proximal AP-1 mutant stable transfectants were generated by co-transfecting HT1080 cells with −670 WT MMP-9 and −670 proximal AP-1 mutant MMP-9 luciferase expression vectors with a selection vector encoding puromycin resistance marker using the Effectene transfection reagent (Qiagen). Stable transfectants were selected by puromycin resistance (0.4 µg/mL) and screened for expression by measuring luciferase activity. Those clones that were confirmed to have luciferase activity were expanded in DMEM, and used for further experiments.

### Transient Transfection and Luciferase Activity Assays

HT1080 cells were transiently transfected in triplicate using Effectene transfection reagent (Qiagen). A plasmid encoding Renilla luciferase (pRL-null, Promega) was co-transfected for the normalization of transfection efficiency, unless otherwise indicated. For experiments where protein concentration was used for normalization, cells were transfected with the indicated MMP-9 contruct as a single pool of cells (in a 100 mm dish). Cells are trypsinized and evenly distributed into the wells of a six-well plate prior to designation of treatment condition. 24 hours after transfection, the cells were treated with 30 nM PMA and 100 U/mL IFNβ for 18 hours prior to preparation of cell extracts, unless otherwise indicated. Cell extracts were assayed for firefly and Renilla luciferase activities using the Dual-Luciferase Reporter Assay system reagent Stop & Glo, and assayed according to manufacturer’s directions (Promega).

### Electrophoretic Mobility Shift Assay (EMSA)

HT1080 cells at 70% confluency were either untreated, treated with PMA (30 nM), or PMA plus IFNβ (100 U/mL) for 30 minutes. Cells were washed in ice-cold PBS and were collected by centrifugation at 600 g at 4°C for 1 minute. Cells were resuspended in 320 µL of hypotonic buffer (10 mM HEPES pH 7.9, 2 mM MgCl_2_, 10 mM KCl, 0.1 mM EDTA, 1 mM DTT, 0.5% IGEPAL, 0.5 mM PMSF) and incubated on ice for 10 minutes. Nuclei were collected again by centrifugation at 12000 g at 4°C for 1 minute and were resuspended in 100 µL of high salt buffer (50 mM HEPES pH 7.9, 300 mM NaCl, 50 mM KCl, 0.1 mM EDTA, 1 mM DTT, 10% glycerol, 0.5 mM PMSF) with slow rotation at 4°C for 30 minutes. After centrifugation at 12000 g at 4°C for 30 minutes, the supernatant was collected as nuclear extract and stored at −80°C for the use of EMSA. For EMSA, 2–5 µg of nuclear extracts were incubated with 1 µL of probe (30,000 cpm) for 20 minutes in 10 µL of binding buffer (10 mM HEPES (pH 7.5), 75 mM KCl, 1 mM dithiothreitol (DTT), 10 µg/mL poly(dI-dC), and 15% glycerol) prior to electrophoresis. Probes corresponding to the proximal AP-1 binding site (upper strand, 5′-CACACCCTGACCCCTGAGTCAGCACTTGCCTGTCAAG-3′), had been labeled with [γ-^32^P]ATP using T4 polynucleotide kinase (New England Biolabs). The resulting DNA-AP-1 complexes were separated on 5% polyacrylamide non-denaturing gels by electrophoresis. Gels were dried and visualized using phosphorimager analysis. Competitive binding analysis was performed following the same protocol, except that samples were also incubated with 100-fold molar excess of specific unlabelled competitor oligonucleotides containing consensus AP-1 binding site (5′-CGCTTGATGAGTCAGCCGGAA-3′), purchased from Promega. Nonspecific competitor oligonucleotides were utilized in similar manner, which were a consensus TFIID sequence (5′-GCAGAGCATATAAGGTGAGGTAGGA-3′).

### Chromatin Immunoprecipitation (ChIP)

Upon determination that the peak of transcriptional repression (IFN) or activation (PMA) of MMP-9 occurs at approx. 12 hrs (IFN) and 4 hrs (PMA) (Ma et al 2004 and authors’ own observations), HT1080 cells were treated with IFNβ for 12 hrs, PMA for 4 hrs, or both. ChIP assays were performed according to Upstate (Millipore) EZ-ChIP instructions. Briefly, cells were formaldehyde crosslinked, then were lysed and chromatin was sonicated to an average size between 400 and 800 bp. Antibodies specific to p65, histone deacetylase (HDAC)-1, or acetylated histone-3 (aH3) (all from Upstate) were used for immunoprecipitations, followed by PCR amplification. PCR products were determined to be in the linear range of amplification at 36–40 cycles. No-antibody negative control immunoprecipitations were performed alongside each independent experiment, and 1% of chromatin was removed from samples prior to immunoprecipitation and subjected to PCR to control for variation in immunoprecipitation starting material. The primers used were as follows:

forward (5′-GCGGTACCGCCCTTTCTCATGCTGGTGCTGCC-3′),

and reverse (5′-GCCTCGAGTGGTGAGGGCAGAGGTGTCT-3′).

In case of stable lines, reverse (5′-GGATAGAATGGCGCCGGGCC-3′) corresponding to the luciferase reporter region. Band intensities were quantified using ImageQuant software (GE Healthcare). Immunoprecipitated bands were normalized to the values of the input bands, and band intensities compared within each immunoprecipitation to the strongest band, represented as 1.

### Statistics

For the analysis of fold induction of luciferase activity of reporter gene expression constructs, a two-tailed Student’s t test was performed with equal variance comparing values as indicated by brackets. A value of *P* < 0.01 was considered statistically significant.

## Supporting Information

Figure S1 **Effect of histone acetylation modifiers on MMP-9 promoter activity.** (A) HDAC1 overexpression results in repression of MMP-9 promoter in a dose-dependent manner. HT1080 cells were co-transfected in 6-well dishes in triplicates with varying amounts of HDAC1 expression construct and −1.2 kb MMP-9 promoter construct. 24 hours after transfection, cells in triplicate wells of a sample were pooled and redistributed in three wells so that transfection efficiency was the same for the three wells of a single sample. 48 hours after transfection, the cells were treated with 30 nM PMA for 18 hours prior to preparation of cell extracts. Cell extracts were assayed for firefly luciferase activities, and normalized by protein concentration to account for any cell concentration differences between samples. Black bars: untreated values, white bars: PMA treated values. The experiment was repeated three times and the error bars represent standard deviation calculated from three separate experiments. The P-values were calculated using statistical analysis software. As indicated above bars, *, **, and symbols indicate P values that indicate significant difference (0.0005, 0.0009, and 0.0006 resp.) with significant values being <0.01. (B) Overexpression of the HAT p300 rescues the repressive actions of IFNβ. HT1080 cells were co-transfected in 6-well dishes in triplicates with -1.2 kb MMP-9 promoter construct, and with or without 200 ng of the p300 expression construct. Cells were prepared as described previously. Cell extracts were assayed for firefly luciferase activities, and normalized by protein concentration to account for any cell concentration differences between samples. Black bars: untreated values considered as one, grey bars: PMA treated, and white bars: PMA and IFNβ treated. The experiment was repeated three times and the error bars represent standard deviation calculated from three separate experiments. The P-values were calculated using statistical analysis software. As indicated above the bars, *symbol indicates a P value of 0.009 (significant difference) and the symbol # indicates a P value of 0.982 (no significant difference), with significant values being <0.01.
(TIF)
