# Supplementary figures and images for: AP-1 Mediated Transcriptional Repression of Matrix Metalloproteinase-9 by Recruitment of Histone Deacetylase 1 in Response to Interferon β
Source: PLoS One. 2012 Aug 6;7(8):e42152. doi: 10.1371/journal.pone.0042152 (PMC3412826; doi:10.1371/journal.pone.0042152)

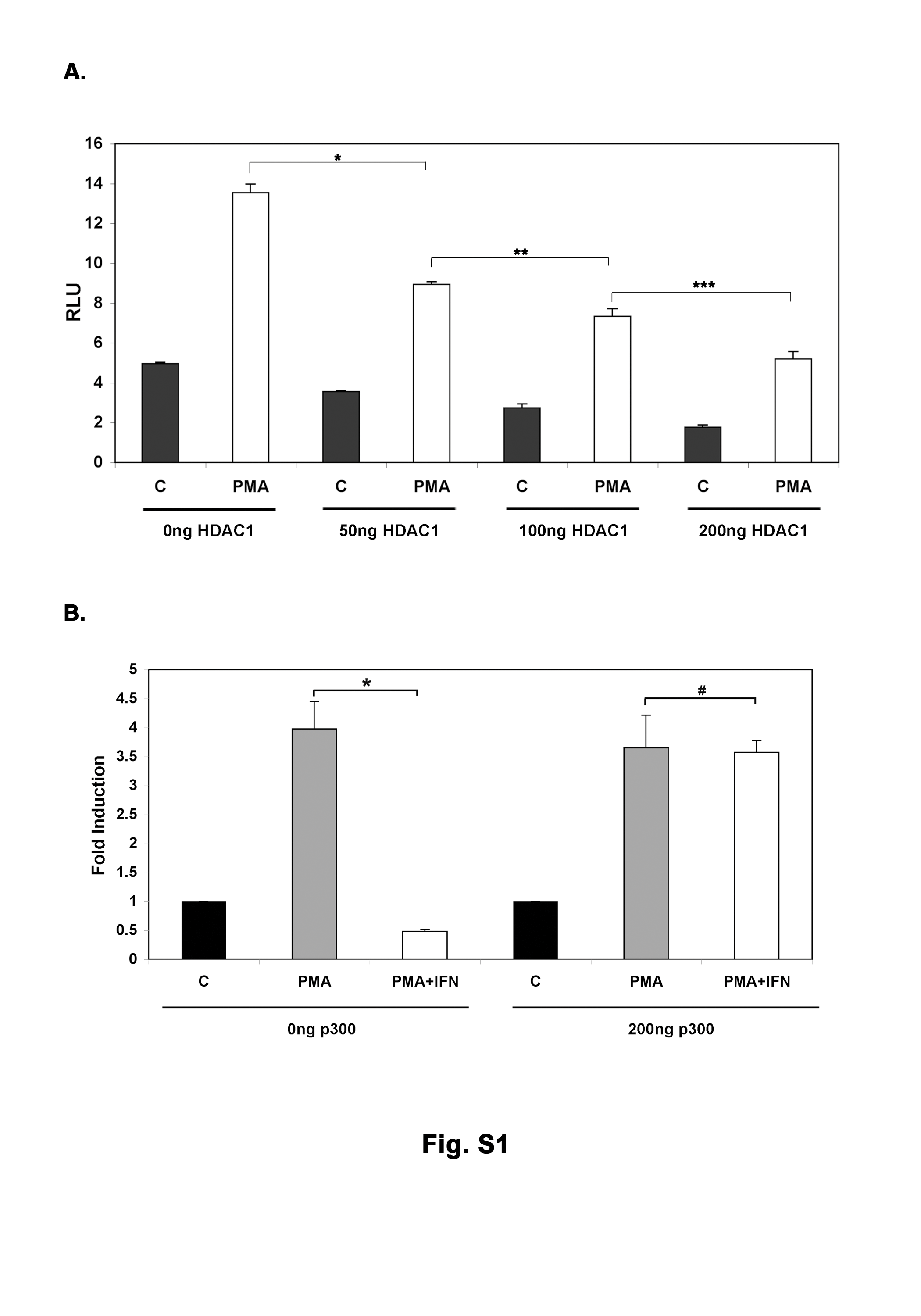

Supplement: Figure S1 — Effect of histone acetylation modifiers on MMP-9 promoter activity. (A) HDAC1 overexpression results in repression of MMP-9 promoter in a dose-dependent manner. HT1080 cells were co-transfected in 6-well dishes in triplicates with varying amounts of HDAC1 expression construct and −1.2 kb MMP-9 promoter construct. 24 hours after transfection, cells in triplicate wells of a sample were pooled and redistributed in three wells so that transfection efficiency was the same for the three wells of a single sample. 48 hours after transfection, the cells were treated with 30 nM PMA for 18 hours prior to preparation of cell extracts. Cell extracts were assayed for firefly luciferase activities, and normalized by protein concentration to account for any cell concentration differences between samples. Black bars: untreated values, white bars: PMA treated values. The experiment was repeated three times and the error bars represent standard deviation calculated from three separate experiments. The P-values were calculated using statistical analysis software. As indicated above bars, *, **, and symbols indicate P values that indicate significant difference (0.0005, 0.0009, and 0.0006 resp.) with significant values being <0.01. (B) Overexpression of the HAT p300 rescues the repressive actions of IFNβ. HT1080 cells were co-transfected in 6-well dishes in triplicates with -1.2 kb MMP-9 promoter construct, and with or without 200 ng of the p300 expression construct. Cells were prepared as described previously. Cell extracts were assayed for firefly luciferase activities, and normalized by protein concentration to account for any cell concentration differences between samples. Black bars: untreated values considered as one, grey bars: PMA treated, and white bars: PMA and IFNβ treated. The experiment was repeated three times and the error bars represent standard deviation calculated from three separate experiments. The P-values were calculated using statistical analysis software. As indi [file pone.0042152.s001.tif]
